# Two-Dimensional Transition Metal Boride TMB_12_ (TM = V, Cr, Mn, and Fe) Monolayers: Robust Antiferromagnetic Semiconductors with Large Magnetic Anisotropy

**DOI:** 10.3390/molecules28247945

**Published:** 2023-12-05

**Authors:** Huiqin Zhang, Nini Guo, Ziyu Wang, Yuqi Xiao, Xiangfei Zhu, Shu Wang, Xiaojing Yao, Yongjun Liu, Xiuyun Zhang

**Affiliations:** 1 College of Physics Science and Technology & Microelectronics Industry Research Institute, Yangzhou University, Yangzhou 225002, China; 2 College of Physics and Hebei Advanced Thin Films Laboratory, Hebei Normal University, Shijiazhuang 050024, China; 3 Key Laboratory of Quantum Materials and Devices (Southeast University), Ministry of Education, Nanjing 200089, China

**Keywords:** transition metal borides, B_12_ icosahedra, antiferromagnets, biaxial strain, first-principles calculations

## Abstract

Currently, two-dimensional (2D) materials with intrinsic antiferromagnetism have stimulated research interest due to their insensitivity to external magnetic fields and absence of stray fields. Here, we predict a family of stable transition metal (TM) borides, TMB_12_ (TM = V, Cr, Mn, Fe) monolayers, by combining TM atoms and B_12_ icosahedra based on first-principles calculations. Our results show that the four TMB_12_ monolayers have stable antiferromagnetic (AFM) ground states with large magnetic anisotropic energy. Among them, three TMB_12_ (TM=V, Cr, Mn) monolayers display an in-plane easy magnetization axis, while the FeB_12_ monolayer has an out-of-plane easy magnetization axis. Among them, the CrB_12_ and the FeB_12_ monolayers are AFM semiconductors with band gaps of 0.13 eV and 0.35 eV, respectively. In particular, the AFM FeB_12_ monolayer is a spin-polarized AFM material with a Néel temperature of 125 K. Moreover, the electronic and magnetic properties of the CrB_12_ and the FeB_12_ monolayers can be modulated by imposing external biaxial strains. Our findings show that the TMB_12_ monolayers are candidates for designing 2D AFM materials, with potential applications in electronic devices.

## 1. Introduction

With the development of spintronics, the search for magnetic semiconductors has become a prominent topic in both scientific and industrial communities due to their great potential to act as next-generation information storage devices. Compared to three-dimensional (3D) magnetic materials, two-dimensional (2D) magnetic materials hold appealing properties such as high crystallinity and flexibility, high optical transparency, large carrier mobility, etc., which are promising in low-power and ultra-compact spintronics at nanoscale [1,2,3,4,5,6,7,8]. Spintronics can combine standard microelectronics with spin-dependent effects, in which the electron spin carries information and provides opportunities for a new platform for designing devices, arising from the interaction between the spin of the carrier and the magnetic properties of the materials. Unfortunately, many 2D materials synthesized in experiments are nonmagnetic, such as graphene [9], silicene [10], phosphorus [11], etc. 

To date, various approaches have been proposed to regulate the spin configurations of 2D materials, such as applying an electric field, imposing external strains, doping, building heterostructures, and so on. However, finding 2D materials with intrinsic magnetism is still a prominent topic and many efforts have been devoted to it. A milestone finding of 2D magnetic materials research is the successful fabrication of an atomically thick CrI_3_ monolayer and a Cr_2_Ge_2_Te_6_ bilayer by exfoliation from their van der Waals bulks, illustrated in 2017 by Gong et al. [12] and Huang et al. [13], respectively [12,13], which highly accelerated the development of 2D magnetic materials. To date, numerous 2D magnetic materials have been reported theoretically or experimentally, such as transition metal (TM) trihalide monolayers (TMX_3_, X = F, Cl, Br, I) [14,15,16,17], TM dichalcogenides or dihalides (TMX_2_, TM = V, Mn, Fe, Co, Ni; X = S, Se, Cl, Br, I) [18,19,20], and ternary materials like Fe_3_GeTe_2_ [21,22,23], TMPX_3_ (X = S, Se, Te) [24,25], TMSiTe_3_ [26,27], CrSnTe_3_ [28], CrGaSe_3_ [29], Cr_2x3_S_3_ (X = Br, I) [30], CrSBr [31,32], EuSn_2_X_2_ (X = P, As) [33], TMInX_3_ (X = Te, Se) [34,35], etc., all of which have been widely studied in terms of magnetoelectric coupling, magnetoresistance, and proximity effects. However, the transition temperatures of most of the known 2D magnetic materials are significantly lower than room temperature, which largely hinders their practical applications. For example, the Curie temperature for the ferromagnetic (FM) bilayer Cr_2_Ge_2_Te_6_ [12], the monolayer CrX_3_ (X = Cl, Br, I) [13,14,15,16,17], and Fe_3_GeTe_2_ [21] are 40 K, 45 K (X = I)/17 K (X = Cl)/34 K (X = Br) and 130 K, respectively. Moreover, compared to FM semiconductors, antiferromagnetic (AFM) semiconductors have become a promising research field due to their robustness against magnetic field perturbation, no stray fields, high-frequency dynamics, etc., which enable them to potentially replace ferromagnets as the active components of spintronic devices [36].

Among the discovered 2D magnetic materials, 2D TM borides composed of TM atoms and boron (B) atoms have attracted great attention in recent years and have become potential candidates for designing 2D magnetic materials. As the valence states of the B atom are unsaturated, it can easily hybridize with exotic atoms and display distinct electronic properties. Currently, a number of 2D TM borides with rich stoichiometries and geometric configurations have been predicted in theory and synthesized in experiments, such as TMB*_x_* (x = 1-6, 8, 9, 12) [37,38,39,40,41,42,43,44,45,46,47], TM*_n_*B*_n_* (n = 1, 2) [37,38,48], TM*_n_*B*_m_* (n = 2, m = 3, 6, 12) [49,50,51], etc., and robust magnetic properties have been found in these TM borides. For example, Ozdemir et al. found that an Fe_2_B_2_ monolayer with rectangular lattice and Fe atoms in different atomic planes has a stable columnar AFM ground state, which can change to an FM order with double hysteresis behavior [48]. Zhang et al. [39] found that the hexagonal FeB_2_ monolayer is a Dirac material with a Fermi velocity comparable to that of graphene. Our group [40] suggested that an FeB_3_ monolayer with a stripe arrangement of Fe atoms is a robust FM metal with a high Curie temperature of 367 K. Zhang et al. [42] predicted the graphene-like FeB_6_ monolayer is an indirect bandgap semiconductor with a band gap of 1.89 eV. In another study, we predicted a type of stable sandwiched B-Fe-B compound, known as the Fe_2_B_6_ monolayer, in which the Curie temperature is as high as 420 K [50]. Recently, we found that the monoatom-thickness FeB_12_ monolayer, composed of a TM©B_8_ wheel and B_4_ cluster within the space group of *Pmmm*, is a stable AFM metal [52]. Therefore, TM borides provide a playground for studying 2D magnetic materials.

Here, based on density functional theory calculations, we report a new class of 2D AFM TM borides, TMB_12_ (TM = V, Cr, Mn, and Fe) monolayers composed of TM atoms and B_12_ icosahedra. Such structures are different from the TMB_12_ monolayer, with the same chemical formula as we predicted previously [52]. On the basis of the results of lattice dynamic calculations and thermodynamic studies, we show that 2D TMB_12_ structures are stable AFM semiconductors or metals. The antiferromagnetism mainly arises from the super-exchange interaction between the B-*p* orbitals and TM-3*d* orbitals. It is found that the VB_12_ monolayer and the MnB_12_ monolayer are AFM metals, while the CrB_12_ monolayer and the FeB_12_ monolayer are AFM semiconductors with band gaps of 0.13 eV and 0.35 eV, respectively. In addition, all four TMB_12_ monolayers have large magnetic anisotropic energy, and three TMB_12_ (TM = V, Cr, Mn) monolayers display an in-plane easy magnetization axis, while the FeB_12_ monolayer has an out-of-plane easy magnetization axis. More interestingly, the AFM semiconducting CrB_12_ monolayer and FeB_12_ monolayer can be modulated into an FM half metal or an FM metal under biaxial strains, and the magnetic transition temperature and band gap can also be adjusted. Our findings provide a new strategy for designing 2D TM borides with potential applications in electronic and spintronic devices, though such TMB_12_ monolayers have not yet been synthesized in experiment. 

## 2. Results and Discussions

**Figure 1:**
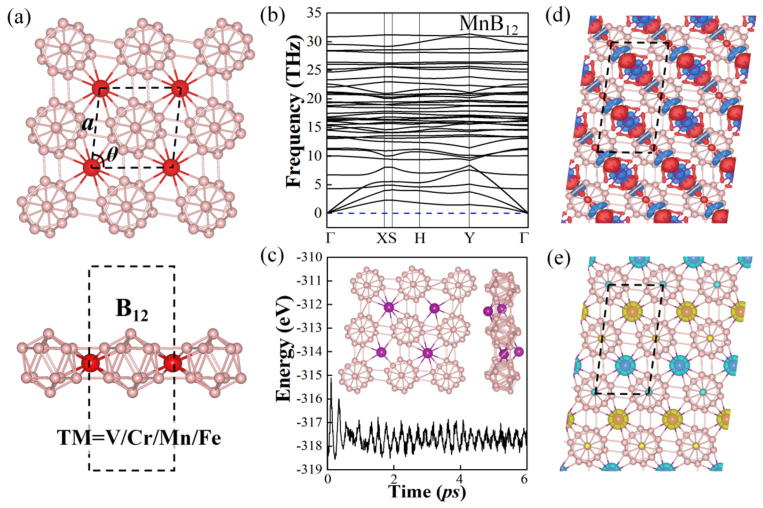
(**a**) Top and side views of the TMB_12_ monolayer; the unit cell is marked by black dotted lines, and red and pink balls represent the TM atoms and B atoms, respectively. (**b**) Phonon spectrum of the MnB_12_ monolayer in the whole Brillouin zone. (**c**) Total energy profiles of the MnB_12_ monolayer and a snapshot of the MnB_12_ monolayer at the end of 6 ps with the temperature of 300 K. (**d**) Charge density difference and (**e**) spin density of the MnB_12_ monolayer. Red and blue colors in charge density differences denote electron accumulation and depletion, respectively. Light blue and yellow regions in the spin density plot represent spin-up and spin-down electrons, respectively.

The top and side views of the TMB_12_ (TM = V, Cr, Mn, and Fe) monolayer are shown in Figure 1a, which consists of TM atoms and B_12_ icosahedra, and the TM atoms bind with four nearby B_12_ icosahedras. The TMB_12_ monolayers have space groups of C2/*m*. Moreover, both phases have the potential to be fabricated by using different precursors in different experimental conditions. As summarized in Table 1, the schematic of lattice constants (a) and monoclinic angles (θ) are shown in the top view of TMB_12_ monolayers in Figure 1a. For TM = V, Cr, Mn, and Fe, the lattice constants are 4.78 Å, 4.78 Å, 4.86 Å, and 4.81 Å, respectively, and the monoclinic angles are in the range of 81.76~83.95°. The TM-B bond lengths for the TMB_12_s are in the range of 1.97~2.35 Å, and the detailed structure parameters of TMB_12_ monolayers are listed in Table 1.

To determine the stability of these TMB_12_ monolayers, phonon spectra and ab initio molecular dynamics (AIMD) calculation curves are calculated. As shown in the phonon dispersion spectra plot in Figure 1b and Figure S1a–c, there are no imaginary frequencies along the high symmetry points path in the whole Brillouin zone, indicating that all the TMB_12_ monolayers are dynamically stable. In addition, the thermodynamical stabilities of the TMB_12_ monolayers were evaluated using AIMD simulations. The total energy profiles of the TMB_12_ monolayer are shown in Figure 1c and Figure S1d–f, and the structures of these TMB_12_ monolayers at the end of 6 ps under 300 K are shown in the insets. It is clearly shown that all the TMB_12_ monolayers could retain their main framework, indicating that they are thermodynamically stable at room temperature. In addition, the formation energy ($E_{f}$) of the TMB_12_ monolayers are calculated using the following Equation (1):

(1) $$E_{f}=(E_{TMB12}-12\mu_{B}-\mu_{TM})/n$$

where $E_{TMB12}$, $\mu_{B}$, and $\mu_{TM}$ are the energies of the TMB_12_ monolayer, the chemical potential of the B atom in the B_12_ icosahedron, and the chemical potential of the TM atom in its bulk, respectively, and *n* is the atom number in the unit cell. The $E_{f}$ values for TM = V, Cr, Mn, and Fe are −0.68, −0.63, −0.74, and −0.58 eV/atom, respectively, implying that the formation of the TMB_12_ monolayers is possible and energetically favorable. It is proposed that the TMB_12_ monolayer may be synthesized by reducing the dimension from bulk to 2D nanosheets [53,54] or by doping the TM atoms in 2D γ-boron film with a certain concentration by the chemical vapor deposition method [55].

The stability of the TMB_12_ monolayers can be explained by the electron transfer between the TM atoms and B_12_ icosahedra. The charge density difference (CDD) plots defined as $\Delta \rho =\rho_{TMB12}-\rho_{TM}-\rho_{B12}$ are calculated and plotted in Figure 1d, where $\rho_{TMB12}$, $\rho_{TM}$, and $\rho_{B12}$ are the electron densities of the TMB_12_ monolayer, the TM atom, and the B_12_ unit, respectively. It is shown that the red regions are distributed around the bonds between the B_12_ icosahedra and the TM atoms, as well as the area near the B atoms on top of the B_12_ icosahedra, while the blue-color regions are mainly around the TM atoms, suggesting that the electrons are depleted from the TM atoms and accumulated on the TM-B bonds and the B atoms of the B_12_ icosahedra, which indicates that the bonding in such complexes is the coexistence of ionic and covalent bonding. Moreover, the electrons transferred between the B_12_ icosahedra and the TM atoms are analyzed by calculating the Bader charge (see Table 1). It is found that about 1.05 e, 0.93 e, 0.93 e, and 0.66 e are transferred from the TM atoms to the B atoms at TM = V, Cr, Mn, and Fe, respectively, which is consist with the charge density difference results in Figure 1d.

**Figure 2:**
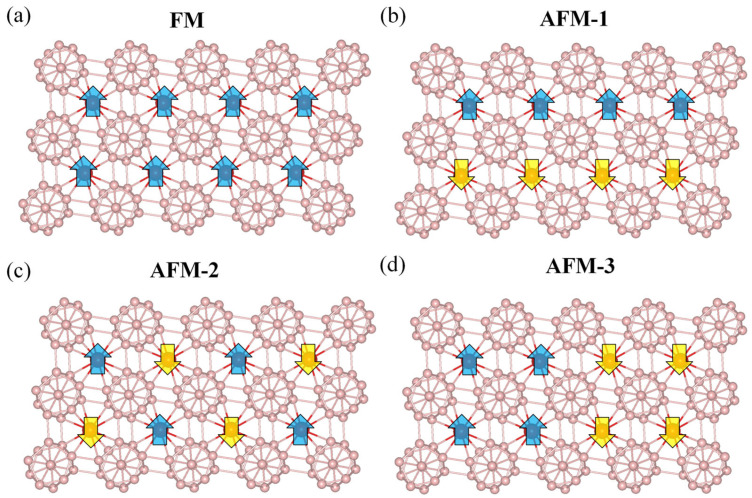
Different magnetic configurations considered for TMB_12_ monolayers. (**a**) FM state, (**b**) AFM-1 state, (**c**) AFM-2 state, and (**d**) AFM-3 state. Blue and yellow arrows are spin-up and spin-down electrons, respectively.

To ascertain the magnetic ground state of the TMB_12_ monolayers, four different magnetic configurations, including one FM state and three AFM states, labeled AFM-1, AFM-2, and AFM-3, are considered, as displayed in Figure 2. (i) AFM-1 configuration: the TM atoms with opposite spins are arranged in a stripy distribution (Figure 2b); (ii) AFM-2 configuration: magnetization with up and down directions are in zigzag distribution (Figure 2c); (iii) AFM-3 configuration: two rows of TM with the same spins are in a stripy arrangement (Figure 2d). According to the energies of these magnetic configurations, it is shown that all the TMB_12_ monolayers favor an AFM ground state, namely, the VB_12_ monolayer has an AFM-2 ground state, which is 0.016 eV/u.c., 0.001 eV/u.c., and 0.011 eV/u.c. lower than those of FM, AFM-1, and AFM-3 states, respectively. The TMB_12_ monolayers with TM = Cr, Mn, and Fe have an AFM-1 ground state, which are lower than the FM, AFM-2, and AFM-3 states by about 0.019~0.086 eV/u.c., 0.001~0.046 eV/u.c., and 0.033~0.093 eV/u.c., respectively. Detailed energy information is summarized in Table S1 in SI. The spin density plots of the TMB_12_ monolayers with different AFM ground states are shown in Figure 1e and Figure S2. The distances of the TM-TM atoms are around 4.78 Å to 4.86 Å, which are much larger than the covalent radius, indicating that the direct exchange interactions are weak. By analyzing the orbital components and the bond angles of the TM-B-TM, it is shown that the antiferromagnetism of the TMB_12_ monolayers mainly arises from the super-exchange interaction, which comes from the *p*-*d* hybridization between the B-*p* orbitals and the TM-3*d* orbitals. According to the Goodenough–Kanamori–Anderson (GKA) rule [56,57,58], systems with TM-B-TM angles close to 90° favor an FM magnetic ordering. As shown in Figure S3 in Supplementary Materials, the two TM-B-TM bond angles are around 119.29–122.16° and 125.73–127.61°, which are much larger than 90°; therefore, antiferromagnetic ordering is preferred through a super-exchange interaction.

**Figure 3:**
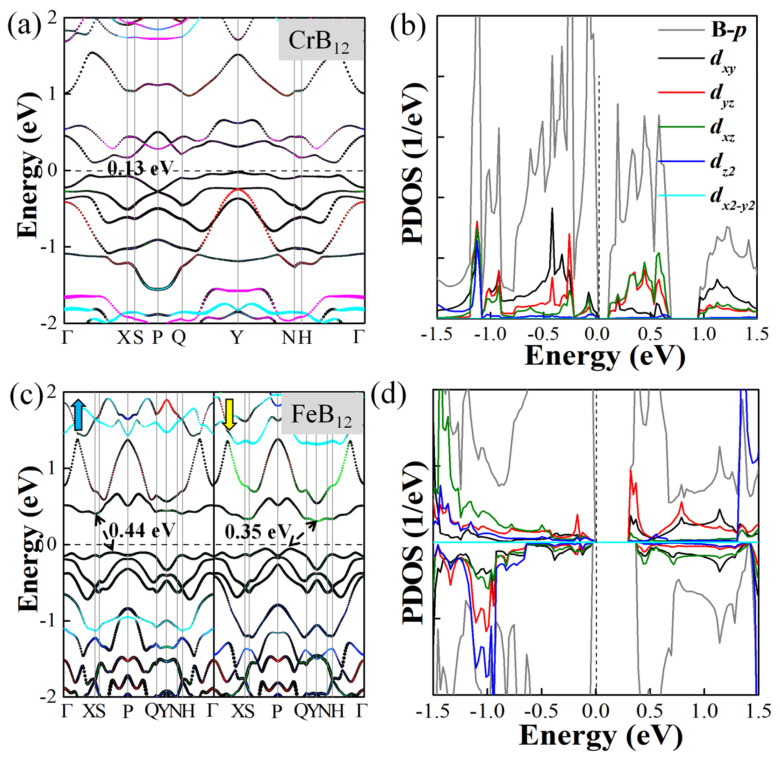
Projected band structure and PDOS of the semiconducting CrB_12_ monolayer (**a**,**b**) and FeB_12_ monolayer (**c**,**d**), respectively. Black, red, green, blue, cyan, and magenta dotted lines are the B-*p*, TM-*d_xy_*, *d_yz_*, *d_xz_*, *d_z2_*, and *d_x2-y2_* orbitals in the projected band structures, respectively. Blue and yellow arrows represent spin-up and spin-down channels, respectively.

The projected band structures of these TMB_12_ monolayers are plotted in Figure 3 and Figure S4. It is shown that the 2D VB_12_ monolayer and the MnB_12_ monolayer are AFM metals with bands crossing the Fermi level. The according projected density of states (PDOS) are also explored, as shown in Figure S4 in Supplementary Materials. For the VB_12_ monolayer, the states around the Fermi level are mainly contributed by the B-*p* and V-*d_z2_* orbitals, while for the MnB_12_ monolayer, the contributions near the Fermi level are mainly from the B-*p* orbitals, and the Mn-*d_yz_*, *d_xz_*, and *d_xy_* orbitals also have a slight contribution. The CrB_12_ monolayer and the FeB_12_ monolayer are AFM semiconductors with band gaps of 0.13 eV and 0.35 eV, respectively. The projected density of states of the semiconducting CrB_12_ monolayer and the FeB_12_ monolayer are plotted in Figure 3b,d. It is indicated that for the CrB_12_ monolayer, the valence band maximum (VBM) is located in the Γ-X path, which is dominated by B-*p* orbitals, while the conduction band minimum (CBM) is located in the Γ-X path, which primarily comes from the hybridization of B-*p*, Cr-*d_xz_*, and Cr-*d_yz_* orbitals. As for the FeB_12_ monolayer (Figure 3d), the CBM located at the X/Y point is dominated by the hybridization of the B-*p*, Fe-*d_xy_*, and Fe-*d_yz_* orbitals, while the VBM located at the S-P/P-Q path comes from the B-*p* orbitals. The magnetic moments of these TMB_12_ monolayers are mainly contributed by the *d* orbitals of TM atoms, while a few are contributed by *p* orbitals of the B atoms. The local magnetic moments on the TM atoms are 2.05 μ_B_, 3.18 μ_B_, 3.86 μ_B_, and 2.79 μ_B_ for TM = V, Cr, Mn, and Fe, respectively.

**Figure 4:**
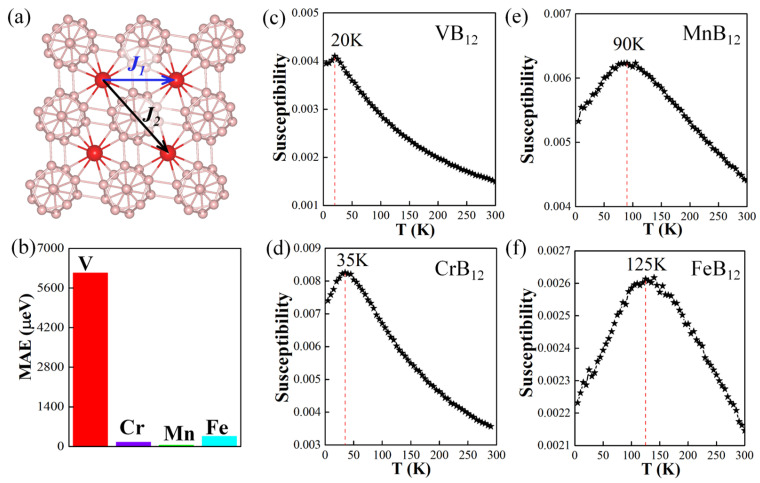
(**a**) Schematic of magnetic configuration for estimating the exchange–interaction constants of TMB_12_ monolayers. *J_1_* and *J_2_* are the nearest and second-nearest exchange parameters, respectively. (**b**) The magnetic anisotropic energy (MAE) of the TMB_12_ monolayers. The susceptibility as a function of temperature for the VB_12_ monolayer (**c**), the CrB_12_ monolayer (**d**), the MnB_12_ monolayer (**e**), and the FeB_12_ monolayer (**f**).

The magnetic anisotropy energy (MAE) is an important parameter of 2D magnets in terms of thermal stability, which is defined as the larger energy difference between the out-of-plane *z* direction and in-plane *x*/*y* directions: MAE = |E_z_ − E_x/y_|. By considering spin–orbit coupling (SOC) effects (the MAE values of the TMB_12_ monolayers are displayed in Figure 4b) it is shown that the easy magnetization axes of the VB_12_ monolayer, the CrB_12_ monolayer, and the MnB_12_ monolayer are in-plane and along the *x* direction, and their MAE values are 6.133 meV, 0.157 meV, and 0.054 meV, respectively. As for the FeB_12_ monolayer, the easy magnetization axis is out-of-plane and along the *z* direction with an MAE of 0.357 meV, which is highly desired for information storage. Furthermore, Monte Carlo (MC) simulations were performed to calculate the T_N_ with the Hamiltonian based on the 2D Heisenberg model written as Equation (2):

(2) $$H=-\sum_{i,j}J_{1}S_{i}\cdot S_{j}-\sum_{i,k}J_{2}S_{i}\cdot S_{k}$$

where *J*_1_ and *J*_2_ are the nearest and the second-nearest exchange coupling parameters as shown in Figure 4a, and ***S_i_*** is the spin vector of TM atom on site *i*. Two exchange parameters (*J*_1_ and *J*_2_) are derived from the energy mapping results of different magnetic configurations as listed in Equation (3):

(3) $$\begin{array}{l} E_{FM}=-(8J_{1}+4J_{2})\times S^{2}\\ E_{AFM-1}=4J_{2}\times S^{2}\\ E_{AFM-2}=-(-8J_{1}+4J_{3})\times S^{2}\\ E_{AFM-3}=-4J_{1}\times S^{2} \end{array}$$

The exchange parameters (*J*_1_ and *J*_2_) can be derived with Equation (4):

(4) $$\begin{array}{l} J_{1}=\frac{E_{2}-E_{0}}{16S^{2}}\\ J_{2}=\frac{2\times (E_{1}-E_{3})-(E_{2}-E_{1})}{16S^{2}} \end{array}$$

The calculated *J*_1_ and *J*_2_ values of the TMB_12_ monolayers according to the energies of different magnetic configurations are listed in Table 1. It is shown that all the *J*_1_ and *J*_2_ values are negative, indicating the AFM coupling between the TM atoms. The susceptibility as a function of temperature for TMB_12_ monolayers are shown in Figure 4c–f. The T_N_s can be obtained by locating the peak of the susceptibility, which are 20 K, 35 K, 90 K, and 135 K for the VB_12_ monolayer, the CrB_12_ monolayer, the MnB_12_ monolayer, and the FeB_12_ monolayer, respectively. Such magnetic transition temperatures are comparable with the reported Cr_2_B_12_ (145 K) and Mn_2_B_12_ (135 K) monolayers [51]. On the other hand, we should state that the magnetic transition temperatures of the predicted systems are much lower than room temperature, which blocks their applications in spintronic devices.

**Figure 5:**
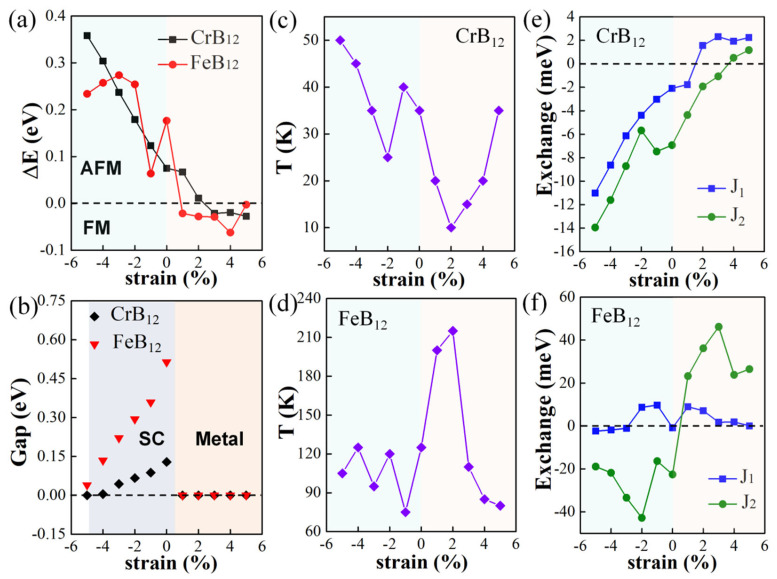
(**a**) The energy difference between FM and AFM state, ΔE = E_FM_ − E_AFM_; (**b**) Band gap of CrB_12_ monolayer and FeB_12_ monolayer under biaxial strains; SC represents semiconductor. (**c**) The variation in magnetic transition temperature (T_C_ for FM configuration/T_N_ for AFM configuration) under biaxial strains for CrB_12_ monolayer (**c**) and FeB_12_ monolayer (**d**). The exchange parameters J_1_ and J_2_ as a function of biaxial strain intensity for the CrB_12_ monolayer (**e**) and FeB_12_ (**f**) monolayer.

Next, the effects of biaxial strains from −5% to +5% on the electronic and magnetic properties of the semiconducting CrB_12_ monolayer and the FeB_12_ monolayer are investigated. The strain intensity is defined as ε = (*a* − *a*_0_)/*a*_0_, where *a*_0_ and *a* are the lattice constant before and after imposing biaxial strains. It is proposed that such strains with a uniform strain gradient may be imposed on the TMB_12_ monolayers by applying a displacement-controlled stretch to the monolayers that can be shaped by focused ion beams (FIB) in experiments. [59] The energy difference (ΔE = E_FM_ − E_AFM_) between the FM and the lowest-energy AFM state as a function of strain intensity are shown in Figure 5a. It is shown that the CrB_12_ monolayer and the FeB_12_ monolayer retain the AFM-1 ground states under biaxial compressive strains, which are transformed to FM ground states when the biaxial tensile strains are up to 3% and 1%, respectively. The band structures of the CrB_12_ monolayer and the FeB_12_ monolayer under biaxial strains are shown in Figures S5 and S6 in Supplementary Materials. It is shown that for the CrB_12_ monolayer, it is turned metallic under tensile strains; that is, it is maintained as an AFM-1 metal under 1% and 2% biaxial tensile strains, but turned to an FM half metal under larger (3~5%) tensile strains with the spin band gap of 0.49 eV, 0.60 eV, and 0.69 eV for ε = 3%, 4%, and 5% (Figure S5c–e in Supplementary Materials), respectively. When exerting biaxial compressive strains, the CrB_12_ monolayer retains the AFM-1 semiconductor property till −4%, with the band gap decreasing with the increasing of the biaxial compressive strains, as shown in Figure 5b and Figure S5f–i. Under −5% biaxial compressive strain, the CrB_12_ monolayer is transformed into an AFM metal (Figure S5j in Supplementary Materials). As shown in Figure S6 in Supplementary Materials, the semiconducting FeB_12_ monolayer transforms into an FM metal under biaxial tensile strain within 4%, and it turns into an FM half metal under 5% tensile strain. When biaxial compressive strains are imposed, it maintains the AFM-1 semiconductor properties, in which the band gap decreases with the increasing of compressive strain as shown in Figure 5b. Moreover, the influence of the magnetic transition temperature of the CrB_12_ and FeB_12_ monolayers under biaxial strains is explored. As shown in Figure 5c,d, Figures S7 and S8, the T_C_ of the FM CrB_12_ monolayer increases with the increasing of the tensile strain from 3% to 5%, and the T_N_ of the AFM CrB_12_ monolayer increases with the increasing of the compressive strain from −2% to −5%. There is a sharp increase in T_N_ at the −2% compressive strain, which is due to the large variation in *J*_2_ as shown in Figure 5e. For the FeB_12_ monolayer, the T_C_ of the FM FeB_12_ monolayer decreases with the increasing of the tensile strains, in which the *J*_1_ and *J*_2_ values are positive. The T_N_ for the AFM semiconductors or metal are oscillating in the range of 75–120 K, which is due to the different changing trends in *J*_1_ and *J*_2_ as shown in Figure 5f.

## 3. Materials and Methods

All the first-principles calculations were performed within the Vienna Ab-initio Simulation Package (VASP) [60,61] with projector-augmented wave (PAW) potentials [62]. The exchange–correlation interaction is described by the general gradient approximation of the Perdew–Burke–Ernzerholf (PBE) functional [63], where the generalized gradient approximation (GGA) was employed. The energy cutoff for the plane–wave basis set was set to 500 eV, and the vacuum spaces were larger than 15 Å to eliminate the physical interactions caused by periodic boundary conditions. For transition metal atoms V, Cr, Mn, and Fe, a GGA + U method with U*_eff_* = 4.0 eV was adopted to treat the Coulomb and exchange interactions of the 3*d*-electron according to previous studies [38,51,64,65]. The *k* mesh was set as 9 × 9 × 1, 5 × 9 × 1, and 5 × 5 × 1 for FM, AFM-1, and AFM-2 geometry optimization, and a denser *k*-mesh of 27 × 27 × 1, 15 × 27 × 1, and 15 × 15 × 1 was adopted for electronic structure calculations. Conjugated gradient (CG) atomic optimization was performed with a criterion of convergence for energy and atom force set to 10^-5^ eV and 0.01 eV/Å, respectively. During the optimization, both the cell shape and the position of the atoms was fully relaxed. The Néel temperature (T_N_) and the Curie temperature (T_C_) of the TMB_12_ monolayers were calculated by using the EspinS package, [66] in which 20 × 20 ×1 lattices were adopted in the Monte Carlo (MC) simulations, and the spins randomly rotated in the space. Phonon dispersions were calculated using 2 × 2 × 1 supercells to reduce the lattice translational constraints using the Phonopy code on the basis of DFPT [67] to identify the dynamic stability, with the threshold for the convergence on energy and force selected as 10^−8^ eV and 10^−4^ eV/Å. The ab initio molecular dynamics (AIMD) simulations in the NVT ensemble were employed to evaluate the thermal stability using 2 × 2 × 1 supercells with a temperature of 300 K for 6 ps using a Nose–Hoover thermostat method [68].

## 4. Conclusions

In summary, we predicted a type of 2D transition metal boride, TMB_12_ (TM = V, Cr, Mn, and Fe) monolayers consisting of transition metal atoms and B_12_ icosahedras, using first-principles calculations. Our results show that all the TMB_12_ monolayers are stable antiferromagnets with large magnetic anisotropy, and the Néel temperatures for the TMB_12_ monolayers range from 20 K to 125 K. Specifically, the CrB_12_ monolayer and the FeB_12_ monolayer are antiferromagnetic semiconductors with band gaps of 0.13 eV and 0.35 eV, respectively. In addition, the electronic and magnetic properties of the semiconducting CrB_12_ monolayer and the FeB_12_ monolayer could be effectively modulated by exerting biaxial strains due to the variation in exchange parameters. In particular, the FeB_12_ monolayer is a spin-polarized antiferromagnet with a Néel temperature of 125 K. By imposing tensile strains, the FeB_12_ monolayer can be altered to be an FM metal with a magnetic transition temperature of up to 210 K. Our findings provide a way to design candidates that are flexible for electronic and spintronic applications.

## Figures and Tables

**Table 1:** The lattice constant (L, Å), monoclinic angle (θ, degree), TM-B bond length (***d***_TM-B_, Å), formation energy (E*_f_*, eV), energy difference between the ground AFM state and the FM state (ΔE = E_FM_ − E_AFM_, eV), local magnetic moment on TM atoms (LMM, μ_B_), electrons transferred from TM atoms to B atoms (Δe, e), exchange integral (*J*_1_, *J*_2_, meV), and Néel temperatures (***T_N_***, K) for TMB_12_ monolayers (TM = V, Cr, Mn, and Fe).

Sys	L	*θ*	*d*_TM-B_	E	ΔE	LMM	Δe	*J*_1_	*J*_2_	*T_N_*
VB_12_	4.78	83.39	2.05–2.30	−0.68	0.07	2.05	1.05	−3.88	−4.81	20
CrB_12_	4.78	83.95	2.06–2.32	−0.63	0.08	3.18	0.93	−2.09	−6.93	35
MnB_12_	4.86	82.52	2.01–2.35	−0.74	0.34	3.86	0.93	−2.80	−16.03	90
FeB_12_	4.81	81.76	1.97–2.34	−0.58	0.18	2.79	0.66	−0.85	−22.58	125

## Data Availability

The data presented in this study are available on request from the corresponding author.

## Supplementary Materials

The following supporting information can be downloaded at: https://www.mdpi.com/article/10.3390/molecules28247945/s1. Figure S1: Phonon spectrum and AIMD simulations of VB_12_, CrB_12_, and FeB_12_ monolayers; Table S1: The energies of TMB_12_ monolayers with FM, AFM-1, AFM-2, and AFM-3 configurations; Figure S2: Spin densities of TMB_12_ monolayers; Figure S3: TM-B-TM bond angles of TMB_12_ monolayers; Figure S4: Projected band structure and PDOS of metallic VB_12_ monolayer and MnB_12_ monolayer; Figure S5: Band structures of CrB_12_ monolayer under biaxial tensile and compressive strains within 5%; Figure S6: Band structures of FeB_12_ monolayer under biaxial tensile and compressive strains within 5%; Figure S7: The susceptibility as a function of temperature for CrB_12_ monolayer under biaxial tensile and compressive strains within 5%; Figure S8: The susceptibility as a function of temperature for FeB_12_ monolayer under biaxial tensile and compressive strains within 5%.
