# Supplementary material for: Two-Dimensional Transition Metal Boride TMB12 (TM = V, Cr, Mn, and Fe) Monolayers: Robust Antiferromagnetic Semiconductors with Large Magnetic Anisotropy
Source: Molecules. 2023 Dec 5;28(24):7945. doi: 10.3390/molecules28247945 (PMC10745289; doi:10.3390/molecules28247945)
Supplement: Supplementary file 1 [file molecules-28-07945-s001.zip › molecules-2678452-supplementary materials.pdf]

Supporting Information

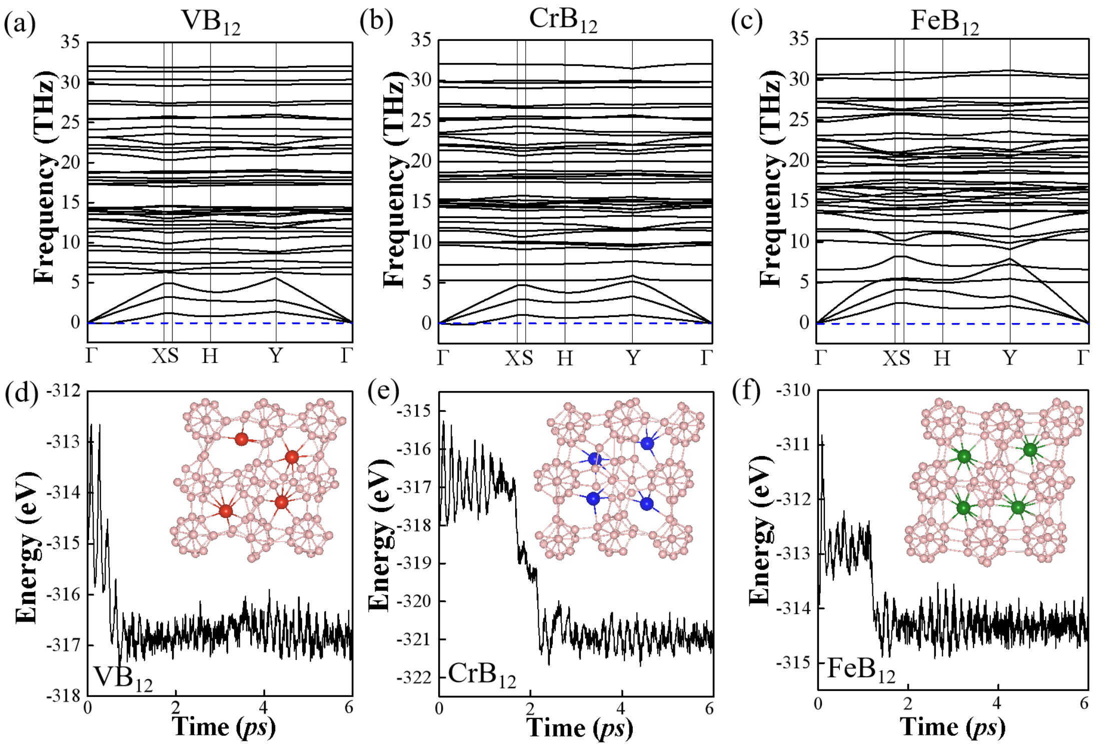

Figure S1. Phonon spectrum of VB_{12} (a), CrB_{12} (b) and FeB_{12} (c) monolayers. Snapshot of VB_{12} (d), CrB_{12} (e) and FeB_{12} (f) monolayer at the end of 6ps with the temperature of 300K.

Table S1. The energies of TMB_{12} monolayers with FM, AFM-1, AFM-2 and AFM-3 configurations. For comparison, the energies of 4*FM and 2*AFM-1, which have the same atoms with AFM-2 and AFM-3 unitcell.

Sys	4*FM	*2*AFM-1*	*AFM-2*	*AFM-3*
VB_{12}	-316.01782556	-316.07598348	-316.07985940	-316.03557312
CrB_{12}	-319.09912712	-319.17423502	-319.16832596	-319.04145682
MnB_{12}	-319.51172856	-319.85668628	-319.67319197	-319.48564614
FeB_{12}	-315.0608248	-315.23731112	-315.08750166	-314.95821819

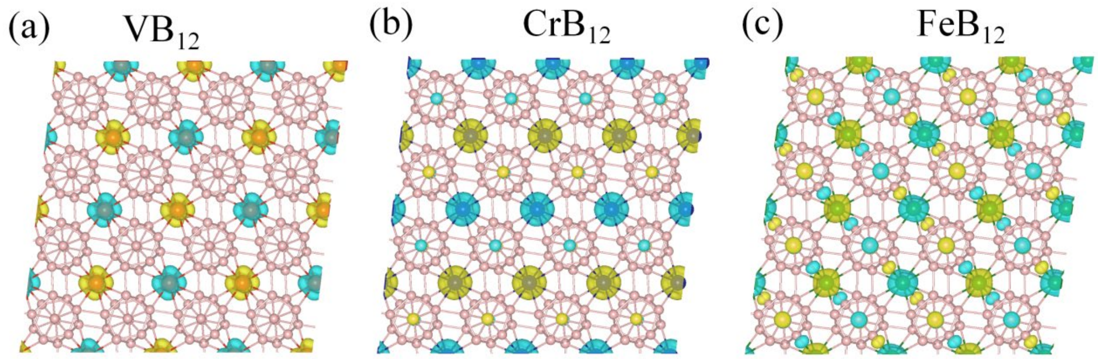

Figure S2. Spin densities of TMB_{12} monolayers for TM=V (a), Cr (b) and Fe (c).

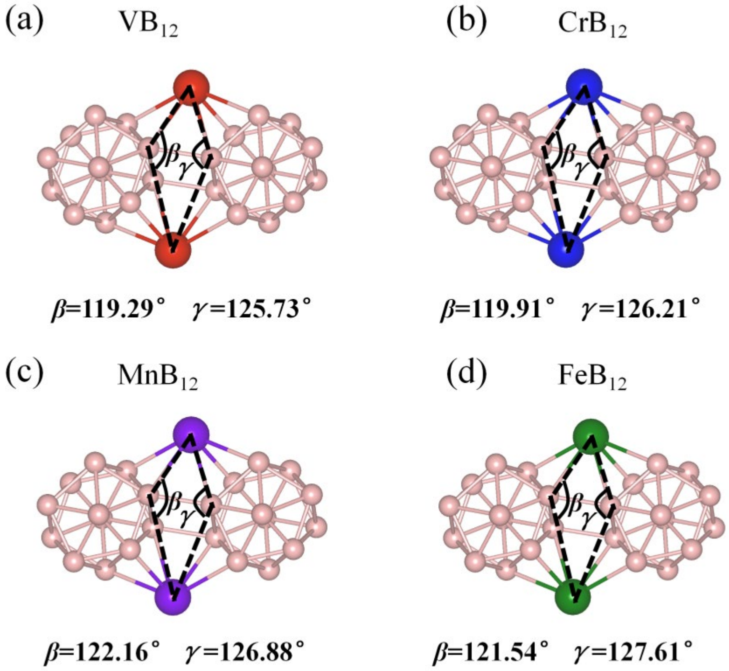

Figure S3. TM-B-TM bond angles of TMB_{12} monolayers for TM=V (a), Cr (b), Mn (c) and Fe (d).

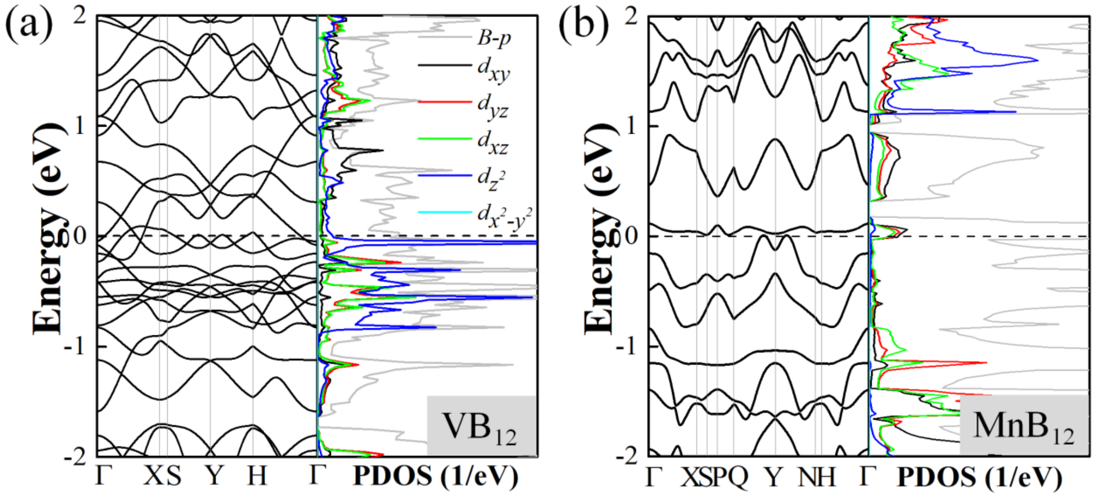

Figure S4. Projected band structure and PDOS of metallic VB_{12} monolayer (a,b) and MnB_{12} monolayer (c,d).

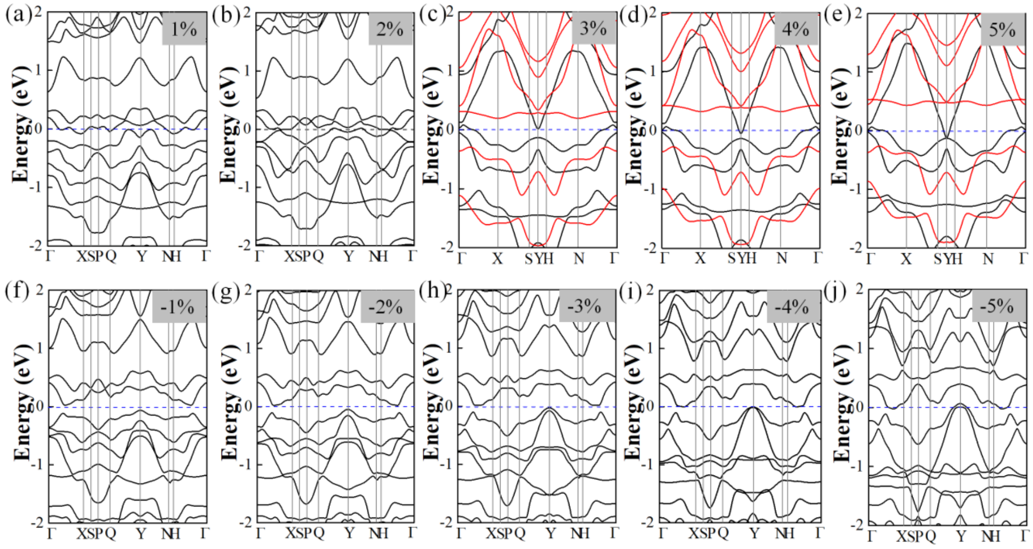

Figure S5. Band structures of CrB_{12} monolayer under biaxial tensile (a-e) and compressive (f-j) strains within 5%.

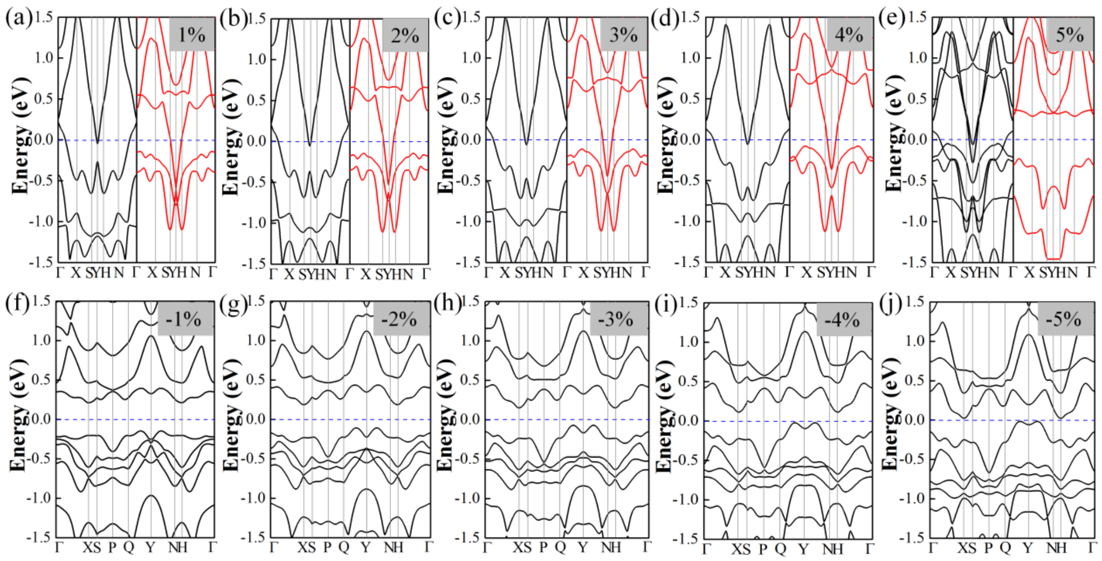

Figure S6. Band structures of FeB_{12} monolayer under biaxial tensile (a-e) and compressive (f-j) strains within 5%.

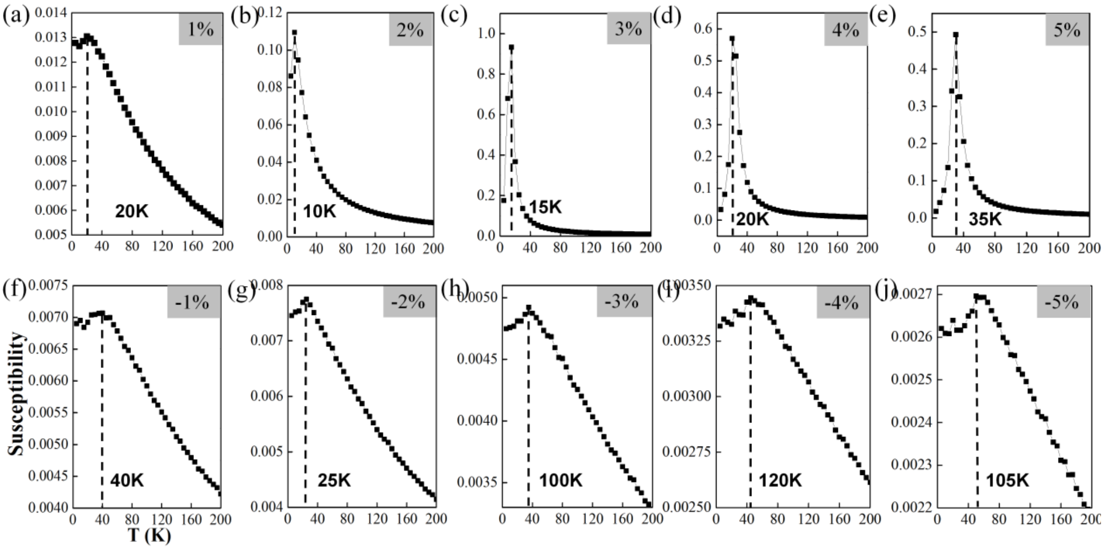

Figure S7. The susceptibility as a function of temperature for CrB_{12} monolayer under biaxial tensile (a-e) and compressive (f-j) strains within 5%.

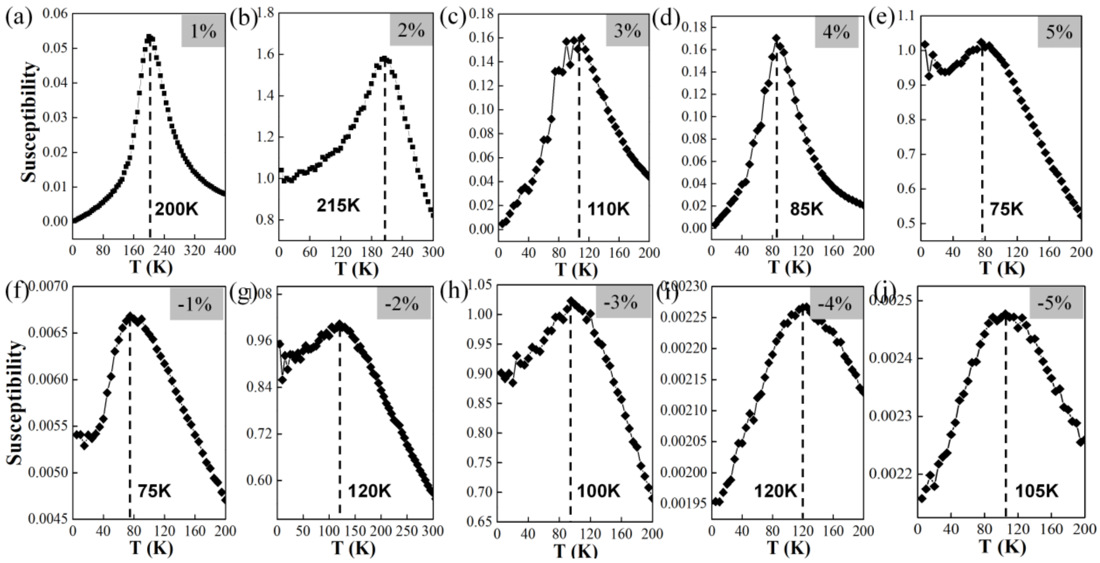

Figure S8. The susceptibility as a function of temperature for FeB_{12} monolayer under biaxial tensile (a-e) and compressive (f-j) strains within 5%.
